# Putting an ‘End’ to HIV mRNAs: capping and polyadenylation as potential therapeutic targets

**DOI:** 10.1186/1742-6405-10-31

**Published:** 2013-12-13

**Authors:** Jeffrey Wilusz

**Affiliations:** 1 Department of Microbiology, Immunology and Pathology, Colorado State University, 80523 Fort Collins, CO, USA

**Keywords:** HIV, Polyadenylation, Capping, 3′ end formation, Post-transcriptional control

## Abstract

Like most cellular mRNAs, the 5′ end of HIV mRNAs is capped and the 3′ end matured by the process of polyadenylation. There are, however, several rather unique and interesting aspects of these post-transcriptional processes on HIV transcripts. Capping of the highly structured 5′ end of HIV mRNAs is influenced by the viral TAT protein and a population of HIV mRNAs contains a trimethyl-G cap reminiscent of U snRNAs involved in splicing. HIV polyadenylation involves active repression of a promoter-proximal polyadenylation signal, auxiliary upstream regulatory elements and moonlighting polyadenylation factors that have additional impacts on HIV biology outside of the constraints of classical mRNA 3’ end formation. This review describes these post-transcriptional novelties of HIV gene expression as well as their implications in viral biology and as possible targets for therapeutic intervention.

## Introduction

Our appreciation of the overall impact and importance of post-transcriptional processes on eukaryotic – and Human Immunodeficiency Virus (HIV) – gene expression has significantly expanded over the last decade. The nuclear processes of pre-mRNA capping, splicing and polyadenylation are now considered largely co-transcriptional in nature and each exerts considerable influence on the transcription process itself [1,2]. Alternative splicing, and to some surprise polyadenylation as well, play a major role in shaping the transcriptome [3,4]. The regulation of the efficiency of nuclear export of HIV transcripts through the Rev/RRE system is well-characterized [5]. Interestingly, recent data suggest a significant amount of ‘two-way’ communication between the stability of an RNA in the cytoplasm and its transcription rate [6,7]. The processes of translation, RNA editing and miRNA-mediated regulation also influence the outcome of HIV gene expression [8-10]. Thus a clear understanding of HIV post-transcriptional events is important for a full appreciation of HIV biology and HIV-host interactions. In addition to their value in understanding basic HIV biology, these new insights into post-transcriptional regulation of HIV gene expression have opened up several novel avenues for possible antiviral therapeutic targeting. Since several aspects of HIV post-transcription control (e.g. splicing, Rev/RRE mediated export, RNA editing) have been the subject of recent reviews [5,9,11], this review will focus on the regulation and impact of HIV mRNA terminal modifications – namely 5′ capping and 3′ polyadenylation - have on HIV gene expression and their potential value as therapeutic targets. Recent insights in these two areas, combined with their fundamental importance to HIV molecular biology, make them rather interesting and attractive processes from both a basic and translational scientific perspective.

### HIV RNA capping – a novel way to put a ‘lid’ on HIV gene expression?

All eukaryotic mRNAs contain a 5′ 7meGpppG ‘cap’ on their 5′ end that is added co-transcriptionally after the first ~20-40 nucleotides of the mRNA are synthesized by RNA polymerase II [12]. Cap addition requires three enzymatic activities – an RNA triphosphatase, a guanyltransferase, and an m^7^guanine methyltransferase – that are present in two proteins that make up the enzymatic components of the human capping enzyme [13] that HIV usurps to cap its own mRNAs. These enzymes are brought to the nascent pre-mRNA by association with the Carboxyl-Terminal Domain (CTD) of the large subunit of RNA Pol II in a phosphorylation-mediated fashion [14]. Interestingly, it has been recently demonstrated that mammalian cells contain a surveillance machinery anchored by the DXO and Xrn2 factors that will rapidly degrade incorrectly capped pre-mRNAs [15,16]. Capping also influences the nuclear processes of transcription [17], splicing [18] and 3′ end formation/polyadenylation [19]. Through interaction with the two proteins of the cap binding complex (CBC), the cap placed on mRNAs driven from the HIV1 promoter up-regulates transcriptional elongation and influences alternative splicing patterns [20]. If one depletes the CBC, TAT- transactivation and transcription elongation are repressed from the HIV1 promoter [21]. In the cytoplasm, the cap is essential for efficient mRNA translation [22] and is a key target for the turnover of mRNAs [23]. Thus it is vitally important for HIV to efficiently cap its mRNAs to maintain a high level of gene expression.

Interfering with the fundamental process of capping of several RNA viruses has been tapped as a potential antiviral target due to the use of viral-derived capping enzymes that bear distinct structures and enzymatic mechanisms [24,25]. This approach is not feasible as an HIV target since the virus utilizes host enzymes to mature the 5′ end of its mRNA. Thus one needs to focus on apparent HIV nuances of the capping process, three of which we believe may pose interesting possibilities as drug targets.

The HIV Tat protein, a small basic intrinsically disordered protein, is well known to interact with the TAR element on the HIV mRNA and recruits transcription factors to promote HIV gene expression [26]. However it is clear that the Tat protein is multi-functional in nature and may also influence RNA interference [27], splicing [28], and notably mRNA capping. The Tat protein stimulates the capping of nascent HIV transcripts by either a direct interaction with the Mce1 component of the human capping enzyme [29,30] or through stimulating the phosphorylation of the CTD of the large subunit of RNA Pol II [31]. Tat also has nucleic acid chaperone activity which may contribute to its ability to stimulate RNA capping [32]. While inhibitors that target Tat interactions as potential anti-HIV therapeutics have been studied for some time e.g. [33-36], perhaps targeting the C-terminal region of Tat that interacts with the Mce1 capping enzyme [30] might be a fruitful approach as well. One potential limitation to this approach, however, is that a firm grasp on the structure of the flexible Tat protein has been elusive.

A second potential therapeutic avenue to follow in HIV capping is the observation that some HIV mRNAs contain 2,2,7 trimethylated guanosine caps instead of the standard ^7^meG cap found on mRNAs [37]. Interestingly, there are several reports that other RNA viruses (a flavivirus and two alphaviruses) also can contain di- and tri-methylated caps on their RNAs [38-40]. Trimethylation of HIV mRNA caps appears to enhance RNA export and improve HIV gene expression [37]. Cap hypermethylation is likely mediated by the cellular PIMT enzyme, a ubiquitously expressed protein that can be found in both the nucleus and cytoplasm of mammalian cells and is best studied for hypermethylation of U small RNA caps involved in splicing [41]. PIMT appears to be recruited to HIV mRNAs via REV/RRE interactions. Overexpression of PIMT can enhance HIV gene expression, while knocking down the enzyme has the converse effect [37]. Interestingly, PIMT activity may be limiting in quiescent cells, and thus be a contributing factor to HIV latency. It is also possible that HIV cap hypermethylation may disrupt U snRNA biogenesis and/or nuclear export. This could impact cellular mRNA splicing, reducing the ability of the HIV infected cells to effectively react to the virus. Thus PIMT and HIV cap hypermethylation may represent an interesting target for therapeutic intervention. Several methylation inhibiting drugs (e.g. the S-adenosyl methionine (SAM) analogue sinefungin, 3-deaza-adenosine and neplanocin A and F analogs) have been shown to decrease HIV replication [37,42-45]. However given the fact that there are numerous enzymes that use SAM and/or methylate cellular substrates, including over 50 lysine methylases, a challenge will be to identify or rationally design small molecule inhibitors that are specific for PIMT. As an alternative strategy, determining ways to increase PIMT activities in quiescent cells may be a viable approach to help drive HIV out of latency and deplete troublesome viral reservoirs. Additional studies to more firmly establish the relationship of PIMT to HIV quiescence would of course be needed to ascertain the likelihood of success with such a strategy. Finally, although highly speculative, this uncommon hypermethylation of HIV caps could be exploited for the specific targeting therapeutics. Since antibodies are capable of specifically recognizing tri-methylated caps, it may be possible to engineer small aptamers that specifically target trimethylated caps in the context of the highly structured 5′ terminal portion of HIV mRNAs.

A final curiosity regarding the 5′ cap is that in both HIV1 and HIV2, the 5′ capped nucleotide is located at the base of a stem in an area of extensive and highly stable secondary structure [46]. In fact, HIV mRNAs require the DDX3 helicase which appears to substitute for the eIF4E cap binding protein to promote translation [47,48]. This structured region may also protect the cap from quality-control surveillance in the nucleus [15] and increase the stability of HIV RNAs by making it difficult for 5′-3′ exonucleases to gain access to an exposed 5′ end. If this is true, then decapped HIV mRNAs may be differentially stabilized in infected cells – and could even be substrates for the recently identified process of cytoplasmic ‘recapping’ of RNAs [49]. Thus small molecules that target aspects of these structures may be useful in reducing HIV gene expression by exposing the cap to normal cellular regulatory controls.

### HIV polyadenylation - can we figure out a way to get an ‘A’ in our course (of treatment)?

The process of 3′ end formation/polyadenylation occurs co-transcriptionally on cellular and HIV mRNAs generated by RNA Pol II and influences the termination of transcription at a site several hundred bases downstream of the mature 3′ end of the mRNA [50]. The 3′ end of most human mRNAs is generated first by an endonucleolytic cleavage event (catalyzed by CPSF73, aka CPSF3) followed by the addition of 100–250 adenylate residues by poly(A) polymerase (PAP). A typical polyadenylation signal contains two types of elements. The core elements consist of an AAUAAA or similar hexanucleotide and a short (about 5 base long) U- or GU-rich tract located within approximately 25–30 bases upstream or downstream, respectively, of the site. The core elements serve as the assembly site of the complex of polyadenylation factors. Many polyadenylation signals also contain auxiliary elements that are located upstream or downstream of the core elements. These auxiliary elements bind to a variety of cellular factors and influence the efficiency of polyadenylation. There are at least 13 core polyadenylation factors and perhaps up to 80 proteins that interact with the complex that forms on the pre-mRNA substrate to generate the mature mRNA 3′ end [51]. This high degree of complexity for the polyadenylation machinery is likely designed to (a) control/target the endonuclease and template independent poly(A) polymerase; (b) network polyadenylation with transcription, capping, splicing and export processes in the nucleus [1,19,52]; and (c) to regulate alternative polyadenylation [53].

Polyadenylation is far from the default process that is typically depicted in textbook descriptions of gene expression. Data generated over the last several years has firmly established the dynamic, highly regulated nature of polyadenylation site choice. Well over 50% of genes are subject to alternative polyadenylation and the process is highly regulated in a tissue-specific and developmentally-specific fashion [53-55]. Altering the site of polyadenylation can truncate protein open reading frames, change splicing patterns or alter mRNA posttranscriptional regulation by shortening the 3′ untranslated region (UTR) and removing sites for miRNA or RNA binding factor interactions [53]. Interestingly, HIV has two polyadenylation signals in its mRNA as a result of the duplicated signal present in the LTR regions [56]. The virus must suppress use of the upstream 5′ polyadenylation site or the short mRNA that is generated will not contain an open reading frame. Additionally, the efficiency of the recognition of the normal 3′ polyadenylation site also has potential pathogenic implications for HIV since the read-though of the normal polyadenylation site is associated with transductive recombination [57,58]. Given the fundamental importance of polyadenylation to HIV gene expression as well the recognition that the process interfaces with numerous nuclear processes and regulatory checkpoints, we believe that at least two aspects of polyadenylation might present possible targets for therapeutic intervention.

A first possible target is the HIV-specific aspects of poly(A) site usage that may in some ways be selectively used by the virus and not the majority of cellular poly(A) signals. Auxiliary elements occur upstream of the normal HIV polyadenylation signal and greatly stimulate its usage. These elements are not located upstream of the 5′ promoter-proximal polyadenylation site due to where transcription begins in the HIV LTR. First suggested by LTR 3′ region deletion experiments performed in the Cullen [59], Ganem [60] and Alwine laboratories [61], the sequence and structural requirements of this 3′ auxiliary element have been extensively studied by Gilmartin and colleagues. Upstream auxiliary sequences that influence HIV polyadenylation appear to include 76 bases upstream of the AAUAAA. This region includes the TAR structural element [26] and importantly a sequence region upstream that collectively assist in the assembly of the core polyadenylation factors, including CPSF and CF1m [62-64] on the downstream polyadenylation region. Given the highly structured nature of this region [46,65,66], it may be possible to develop small molecule inhibitors to disconnect this upstream enhancer function from the HIV polyadenylation signal, resulting in a dramatic decrease in HIV gene expression. RNA structures are viable drug targets as, for example, numerous antibiotics target RNA-derived structures in the ribosome and branched boronic peptides have recently been shown to target the HIV RRE [67]. Alternatively, work in the Proudfoot and Cochrane laboratories has suggested a clear association in the efficiency of HIV polyadenylation and the major 5′ splice site [68,69]. Therefore drugs that influence splicing factors/RNA splicing may have some desirable consequences on HIV gene expression. To date, several studies have presented mixed results targeting a U1 snRNP-based polyadenylation/splicing related inhibition approach to HIV therapy [70,71]. Thus more work is clearly needed in this area. Next, while promoter proximity of the HIV upstream 5′ polyadenylation site clearly represses its activity [60,72], a recent study has demonstrated that activating a cryptic polyadenylation site near promoters can decrease transcriptional activity [73]. Thus determining ways to de-repress the promoter-proximal HIV poly(A) site may yield huge therapeutic dividends. Finally Goff and colleagues have demonstrated using genetic screens and other analyses that HIV polyadenylation is directly regulated by eIF3f, CDK11, and splice factor 9G8 [74]. Thus compounds that regulate a variety of cellular proteins may be capable of repressing HIV polyadenylation and produce some clinical benefit if effects on host cell metabolism can be minimized.

The second possible area of HIV polyadenylation-related therapeutic development may lie in a bevy of unexpected roles for polyadenylation factors that have recently been demonstrated in HIV biology. It is becoming clearer in the field of post-transcriptional control of gene expression that many processes are networked and factors can appear to be ‘moonlighting’ to perform a variety of functions in the cell. Along these lines, CPSF6 (aka CF1m68kd) has been shown to have isoforms that bind HIV capsid protein and regulate HIV disassembly and trafficking to the nucleus [75-77]. Thus small molecules that stabilize or promote the formation of cytoplasmic CPSF6 isoforms may have significant impact on multiple aspects of HIV biology. CPSF3 (aka CPSF73) has been demonstrated to be up-regulated by TAT and repress the HIV promoter [78,79]. Hence targeting this factor could have some impact on driving HIV out of latency in reservoir sites. The polyadenylation and transcription termination factor Pcf11 has been shown to be a negative elongation factor for HIV [80]. Thus, stabilizing or increasing the activity of this protein may have therapeutic benefits. Finally, hyperphosphorylation of poly(A) polymerase (PAP) has been shown to be associated with HIV Vpr expression [81]. Thus this may need to be considered when analyzing the effect of various kinase inhibitors on HIV.

## Conclusion

Targeting host rather than viral-specific factors that influence HIV replication and gene expression is one approach to reduce the likelihood of viral drug resistance. RNA interference based screens have identified a plethora of potential host targets for HIV drug development. While capping and polyadenylation are often considered to be simple default processes in eukaryotic gene expression, numerous studies have made it clear that they are deeply networked and contain HIV-specific nuances that might be considered as possible targets for therapeutic intervention. Given the significance of HIV infection in the world population, we believe that no stones that are revealed by basic science should be left unturned by those searching for novel effective treatments.

## Competing interests

The author declares that he has no competing interests.
